# miR-424-5p promotes proliferation of gastric cancer by targeting Smad3 through TGF-β signaling pathway

**DOI:** 10.18632/oncotarget.12092

**Published:** 2016-09-17

**Authors:** Song Wei, Qing Li, Zheng Li, Linjun Wang, Lei Zhang, Zekuan Xu

**Affiliations:** ^1^ Department of General Surgery, The First Affiliated Hospital of Nanjing Medical University, Nanjing, Jiangsu, China

**Keywords:** miR-424-5p, Smad3, proliferation, gastric cancer, TGF-β signaling pathway

## Abstract

MiRNAs have been reported to regulate gene expression and be associated with cancer progression. Recently, miR-424-5p was reported to play important role in a variety of tumors. However, the role and molecular mechanisms of miR-424-5p in GC (gastric cancer) remains largely unknown. In this study, we aimed to explore the role of miR-424-5p in GC. QRT-PCR was used to determine the expression levels of miR-424-5p and Smad3. CCK8 assay, plate clone assay and cell cycle assay were used to measure the effects of miR-424-5p on GC cell proliferation. Luciferase reporter assay and western blotting were used to prove that Smad3 was one of the direct targets of miR-424-5p. Tumorigenesis assay was used to investigate the role of miR-424-5p in tumor growth of GC cells *in vivo*. We found that miR-424-5p was up-regulated in GC tissues and cells. Over-expression of miR-424-5p could promote the proliferation of GC cells. In addition, luciferase reporter assay and western blotting assay revealed that Smad3 was a direct target of miR-424-5p. Over-expression of Smad3 could partially reverse the effects of miR-424-5p on GC cell proliferation. Our study further revealed that miR-424-5p could inhibit TGF-β signaling pathway by Smad3.

## INTRODUCTION

Gastric cancer (GC) is the third most common cause of tumor-associated death in the world [1]. Almost half of the gastric cancer patients were Chinese, most of them were diagnosed with advanced stage [2]. Treatments of gastric cancer include surgery, postoperative chemotherapy, adjuvant chemotherapy and radiotherapy [3]. Although advancement in these treatments, the prognosis for patients of gastric cancer remains poor [4]. Therefore, detection of molecular targets for gastric cancer to improve prognosis of GC patients is particularly important [5].

MiRNAs are endogenously short (18-25 nucleotides) non-coding RNA [6]. miRNAs lead to the alteration of mRNA through binding to the 3' untranslational region (3'UTR) of their target genes. The role of miRNAs includes two categories, tumor suppressors that inhibit cell proliferation and oncogenes that promote cell proliferation [6]. The exact mechanisms how miRNAs regulate their target genes are not fully understood [7]. It has been reported that miR-424-5p was dysregulated in many tumors. For example, miR-424-5p was up-regulated in pancreatic cancer [8] and colon cancer [9]. While miR-424-5p was down-regulated in esophageal squamous cell carcinoma [10] and non-small cell carcinoma [11]. However, little is known about the role of miR-424-5p in gastric cancer. In this study, we found that miR-424-5p was up-regulated in gastric cancer and promoted GC cell proliferation both *in vitro* and *in vivo*.

It has now been confirmed that the alteration of Smad3 expression could cause the occurrence and development of cancer [12]. Han et al have reported that Smad3 functions as a tumor suppressor in gastric cancer [13]. Smad3 have been identified as a critical member in TGF-β signaling pathway [14]. It has been reported that TGF-β strongly inhibits the growth of many tumor cells in the early stage of tumor progression [15]. In this study, we aimed to explore the role of miR-424-5p in gastric cancer. Through bioinformatics predication and experiments confirmation, we found that Smad3 was one of the direct targets of miR-424-5p. Over-expression of Smad3 could partially reverse the effects of miR-424-5p on GC cell proliferation.

## RESULTS

### MiR-424-5p is up-regulated in gastric cancer tissues and cells

**Figure 1:**
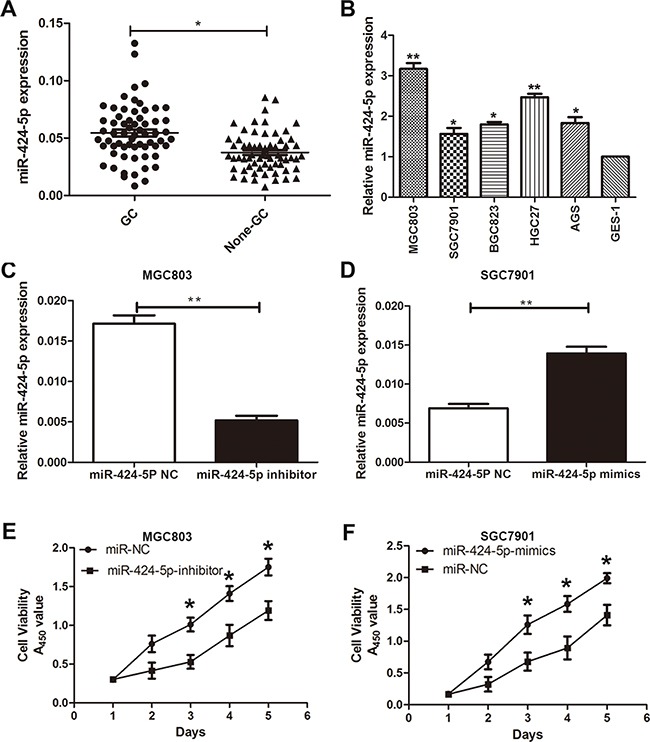
MiR-424-5p was up-regulated in GC tissues and cells **A.** The expression levels of miR-424-5p in 63 pairs of human GC tissues and adjacent normal tissues by qRT-PCR. **B.** The expression levels of miR-424-5p in GC cells and GES-1. **C.** and **D.** miR-424-5p expression in cells transfected with miR-424-5p inhibitor and miR-424-5p mimics lentivirus. **E.** and **F.** Cells transfected with miR-424-5p inhibitor and miR-424-5p mimics lentivirus were determined using a CCK8 assay.

The expression level of miR-424-5p was examined in 63 pairs of GC tissues and adjacent normal tissues by qRT-PCR. As shown in Figure 1a, the expression level of miR-424-5p was up-regulated in GC tissues. We further examined miR-424-5p expression in GC cells and GES-1 by qRT-PCR. As shown in Figure 1b, the expression level of miR-424-5p was significantly higher in GC cells than that in GES-1. In addition, we have analyzed the correlation between the expression levels of miR-424-5p or Smad3 and clinicopathological features of GC patients. We divided GC patients into two groups according to the expression of miR-424-5p or Smad3. GC tissues with higher than the median expression of miR-424-5p or Smad3 were selected into high group, while those with less than the median expression of miR-424-5p or Smad3 were selected into low group. As shown in Table 1, the expression level of miR-424-5p was up-regulated in the tumor size larger than 3cm group. However, Smad3 expression in this group showed the opposite results.

**Table 1:** Expression of miRNA-424-5p expression and Smad3 in human gastric cancer according to patients' clinicopathological characteristics

Characteristics	Number (%)	miR-424-5p expression		P-value	Smad3 expression		P-value
		High group	Low group		High group	Low group	
Age(years)							
<60	18	7	11	0.380	10	8	0.425
≥60	45	23	22		20	25	
Gender							
Male	29	16	13	0.268	14	15	0.023
Female	34	14	20		16	18	
Size(cm)							
<3	30	9	21	0.008*	20	10	0.004*
≥3	33	21	12		10	23	
Histology grade							
Well-moderately	19	11	8	0.283	10	9	0.601
Poorly-signet	44	19	25		20	24	
stage							
I/II	22	9	13	0.435	8	14	0.190
III/IV	41	21	20		22	19	
T grade							
T1+T2	20	8	12	0.058	7	13	0.171
T3+T4	43	22	21		23	20	
Lymph node metastasis							
Present (N1-N3)	40	22	18	0.122	16	24	0.110
Absent(N0)	23	8	15		14	9	

* p<0.05 Statistically significant difference

### MiR-424-5p promotes the proliferation of gastric cancer cells

**Figure 2:**
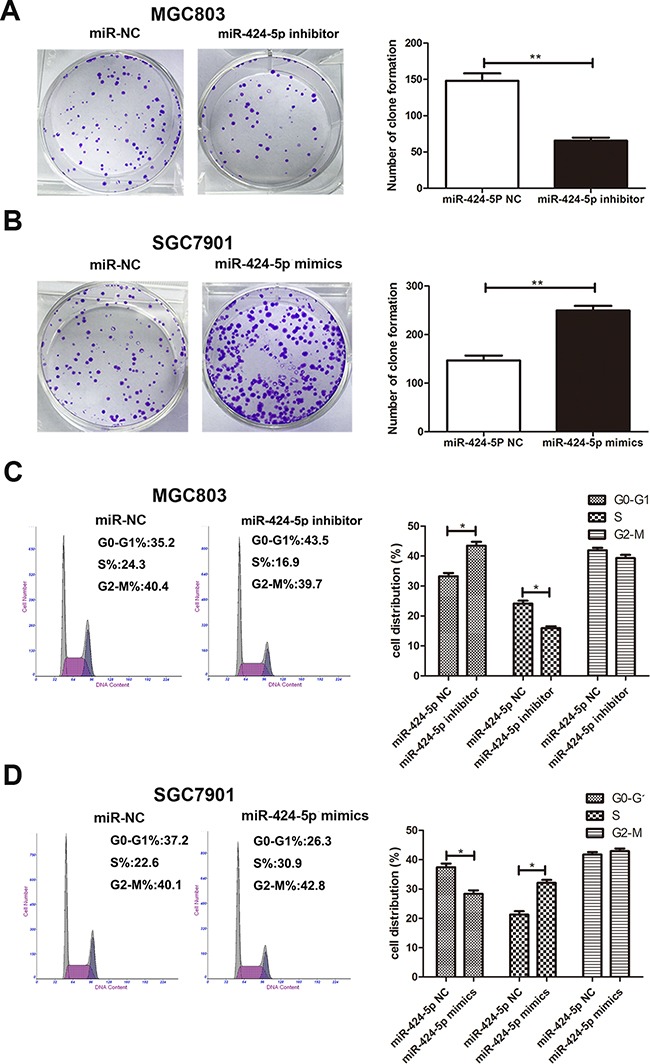
Effects of miR-424-5p expression on GC cell proliferation **A.** and **B.** The colony formation results of cells transfected with miR-424-5p inhibitor and miR-424-5p mimics lentivirus. **C.** and **D.** The effects of miR-424-5 inhibitor or mimics on cell cycle distribution of GC cells.

To further investigate the role of miR-424-5p in gastric cancer. Based on the results of expression levels of miR-424-5p in GC cells by qRT-PCR, MGC803 and SGC7901 cells were transfected with miR-424-5p inhibitor or mimics lentivirus respectively. We then performed qRT-PCR to verify the efficacy of transfection (Figure 1c and 1d). CCK-8 assay was used to examine the affection of miR-424-5p on the proliferation ability of GC cells. The results revealed that the growth rate of MGC803 cells transfected with miR-424-5p inhibitor was significantly decreased compared with control, while SGC7901 cells transfected with miR-424-5p mimics showed the opposite effects (Figure 1e and 1f). Consistently, colony formation assay showed that over-expression of miR-424-5p could promote GC cell proliferation, whereas knockdown of miR-424-5p could reverse these effects (Figure 2a and 2b). Using cell cycle assay, the cell cycle distribution of cells was determined by fluorescence-activated cell sorting (FACS) analysis. We have discovered that MGC803 cells transfected with miR-424-5p inhibitor showed a significant increase in the percentages of cells in the G0/G1 phase (Figure 2c). However, SGC7901 cells transfected with miR-424-5p mimics showed the different affection (Figure 2d). In general, our results revealed that over-expression of miR-424-5p promoted the proliferation of gastric cancer cells *in vitro,* knockdown of miR-424-5p could induce cell cycle arrest in G0/G1 phases.

### Smad3 is down-regulated in human gastric cancer tissues and cells

**Figure 3:**
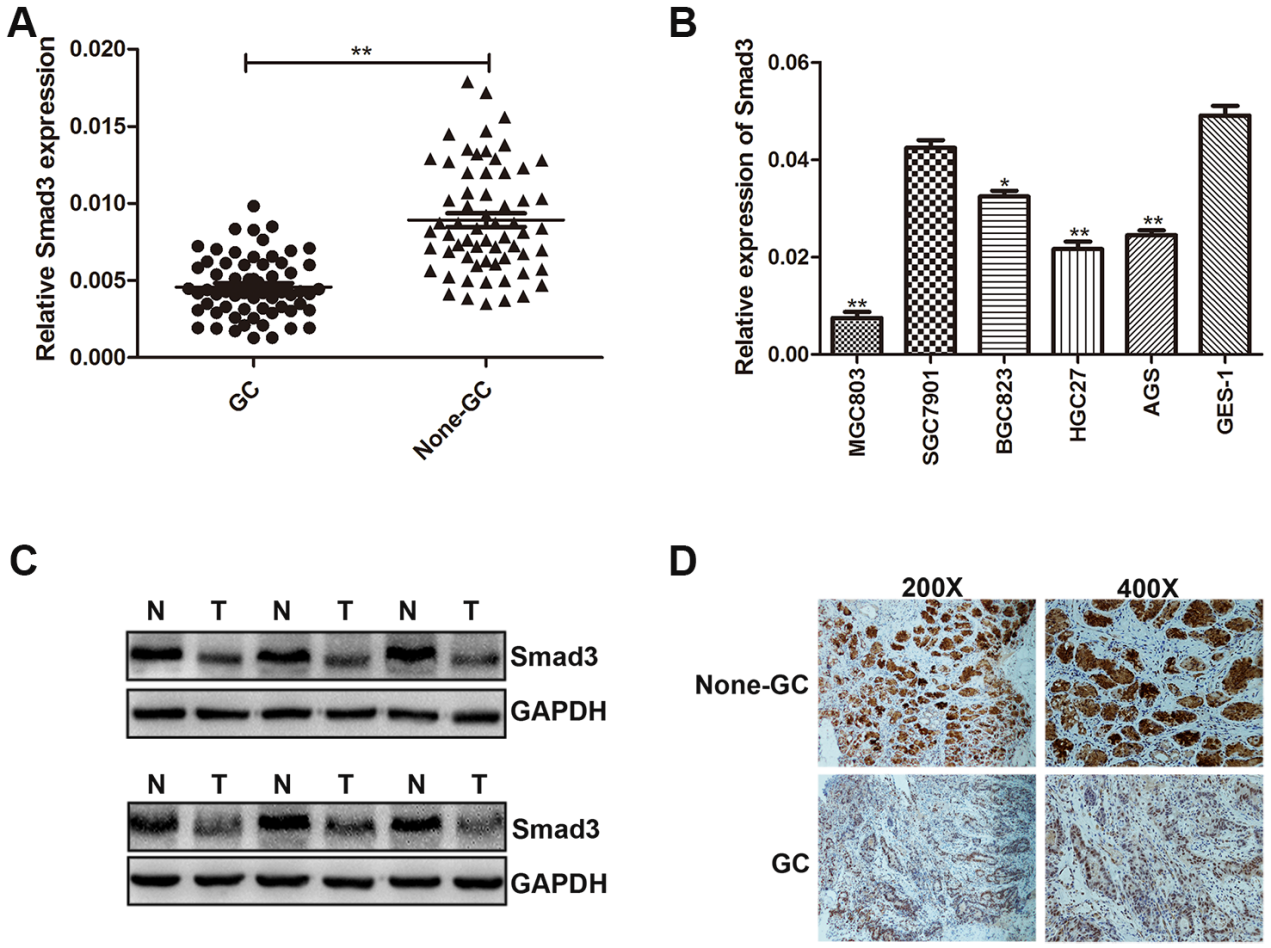
Smad3 was down-regulated in GC tissues and cells **A.** The expression level of Smad3 was determined in 63 pairs of human GC tissues and adjacent normal tissues by qRT-PCR. **B.** The expression level of Smad3 in GC cells and GES-1. **C.** Smad3 protein level was examined by western blotting in six paired of GC tissues. **D.** Smad3 protein level in GC specimens and adjacent normal tissues was determined by immunohistochemistry staining.

In order to examine the association between miR-424-5p and Smad3, we have analyzed the expression level of Smad3 in 63 paired human GC specimens and adjacent normal tissues by qRT-PCR at first. As shown in Figure 3a, the expression level of Smad3 was down-regulated in GC tissues. QRT-PCR was used to determine the expression level of Smad3 in GC cell lines and GES-1. We have discovered that Smad3 had a lower expression in GC cell lines than GES-1 (Figure 3b). We next examined the Smad3 expression in six paired GC tissues by western blotting. As shown in Figure 3c, the expression level of Smad3 was lower in GC tissues than that in adjacent normal tissues (Figure 3c). Consistently, we also found that Smad3 was down-regulated in GC tissues via immunohistochemistry (Figure 3d).

### Smad3 was a direct target of miR-424-5p

**Figure 4:**
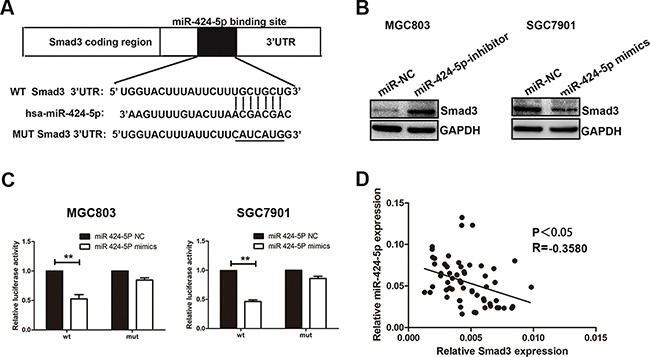
Smad3 was a direct target of miR-424-5p **A.** The potential miR-424-5p binding site at the 3'-UTR of Smad3 mRNA was computationally predicted by Tragetscan. **B.** Smad3 protein level in GC cells transfected with miR-424-5p inhibitor lentivirus and miR-424-5p mimics lentivirus. **C.** Luciferase activity was analyzed in cells co-transfcted with miR-424-5p mimics or negative control with pGL3-Smad3 or pGL3-Smad3-mut. **D.** A negative correlation between the expression levels of miR-424-5p and Smad3 in GC specimens (P<0.05).

Through the miRNA target prediction websites (starBase, Targetscan and miRanda), we found that Smad3 might be one of the target genes of miR-424-5p (Figure 4a). To demonstrate the computational prediction results, western blotting was used to determine the expression of Smad3 protein after the changes of miR-424-5p expression. As shown in Figure 4b, we found that over-expression of miR-424-5p could down-regulate the Smad3 protein expression, whereas knockdown of miR-424-5p showed the opposite results. We further explored whether miR-424-5p could directly target the 3'-UTR of Smad3 mRNA by luciferase reporter assay. We have cloned the 3'-UTR fragment with target sequence into the pGL3 luciferase reporter vector (pGL3-Smad3). 3'-UTR fragment with mutated sequence was also cloned into pGL3 luciferase reporter vector as a control (pGL3-Smad3-mut). We have noticed that co-transfection with miR-424-5p mimics and the pGL3-Smad3 vector showed a significantly decreased luciferase activity in MGC803 and SGC7901 cells. However, the luciferase activity of the same cells transfected with pGL3-Smad3-mut vector has not been affected by over-expression of miR-424-5p (Figure 4c). We also found that there was a negative correlation between the expression levels of miR-424-5p and Smad3 in GC specimens (2-tailed Spearman's correction, r=−0.3580, P<0.05) (Figure 4d). In summary, these data suggested that Smad3 gene might be one of the direct targets of miR-424-5p.

### Over-expression of Smad3 could partially reverse the effects of miR-424-5p on GC cell proliferation

**Figure 5:**
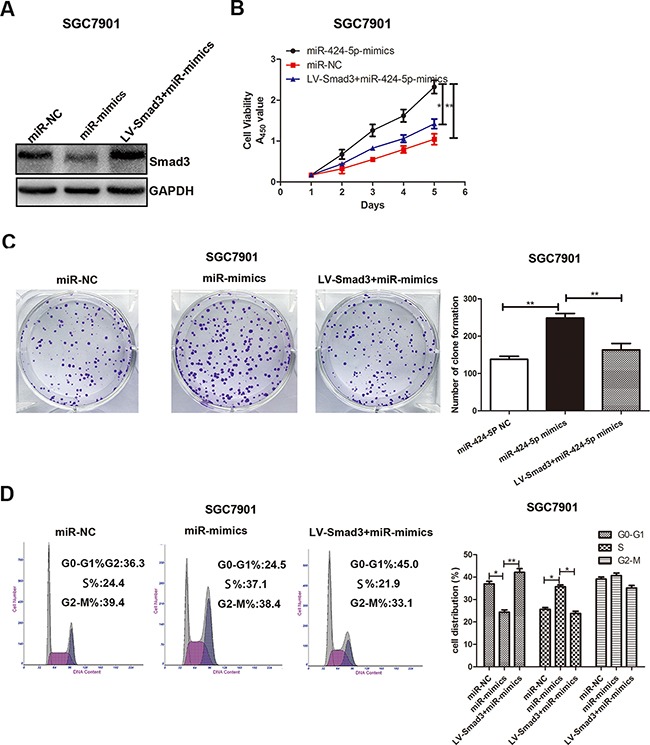
Over-expression of Smad3 could partially reverse the effects of miR-424-5p on GC cell proliferation **A.** The Smad3 protein expression level was measured by western blotting. **B.** Cell growth rate was measured by CCK8 assay. **C.** The colony formation results of GC cells. **D.** The cell cycle distribution of GC cells was measured by flow cytometry.

**Figure 6:**
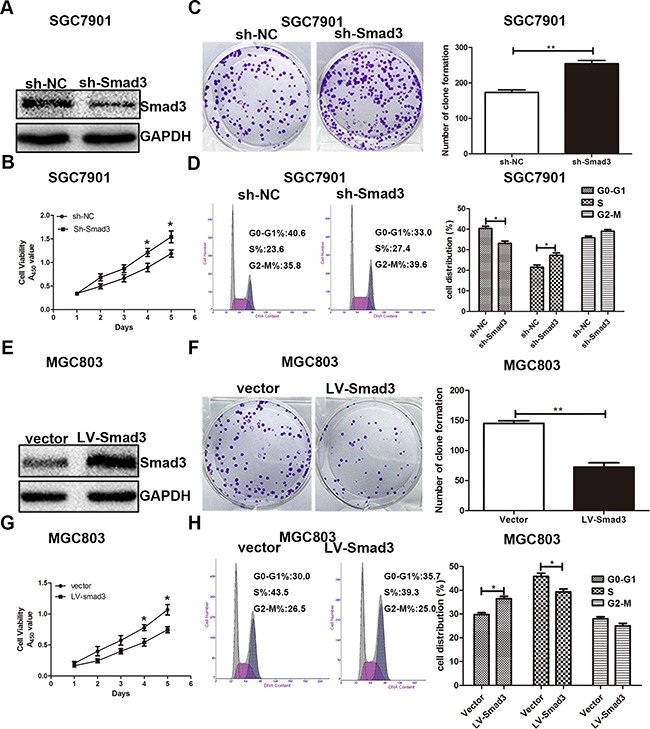
Effects of Smad3 expression on GC cell proliferation **A.** the Smad3 expression in cells transfected with Smad3 inbibitor. **B, C.** and **D.** Cells transfected with Smad3 inhibitor were determined using CCK8 assay, plate clone assay and cell cycle assay. **E.** the Smad3 expression in cells transfected with Smad3 lentivirus. **F, G.** and **H.** CCK8 assay, plate clone assay and cell cycle assay were used to determine the effects of over-expression Smad3 in GC cell proliferation.

To explore whether the effect of miR-424-5p on GC cell proliferation was mediated by Smad3, SGC7901 cells were transfected with miR-424-5p mimics lentivirus for 72h, followed by transfected with LV-Smad3. Over-expression of Smad3 was verified by western blotting (Figure 5a). Through CCK8 assay and plate clone assay, we found that over-expression of Smad3 could significantly reverse the promoting effect of miR-424-5p in SGC7901 cells (Figure 5b and 5c). In order to prove that miR-424-5p induced the loss of Smad3 resulted in the decreased percentage of G0/G1 phase, flow cytometry was used to determine the distribution of cell cycle in cells transfected with miR-424-5p mimics and LV-Smad3. As shown in Figure 5d, over-expression of Smad3 could significantly induce the increased percentage of G0/G1 phases in SGC7901 cells transfected with miR-424-5p mimics. To further verify the effects of promoting the proliferation of GC cells of miR-424-5p over-expression were meditated by inhibiting the expression of Smad3, we have inhibited the expression of Smad3 alone to examine the effects of Smad3 on GC cell proliferation in SGC7901 cells by using shRNA (Figure 6a). As shown in Figure 6b,c and 6d, the CCK8 assay, plate clone assay and the cell cycle assay revealed that knockdown of Smad3 could promote the proliferation of GC cells, which was consistent with the affection of miR-424-5p over-expression on GC cell proliferation. Furthermore, we have also over-expressed the expression of Smad3 in MGC803 cells which were transfected Smad3 lentivirus (Figure 6e). It was shown that over-expression of Smad3 could inhibit the proliferation of GC cells (Figure 6f,g and 6h).

### MiR-424-5p promotes tumor growth of GC cells in nude mice

**Figure 7:**
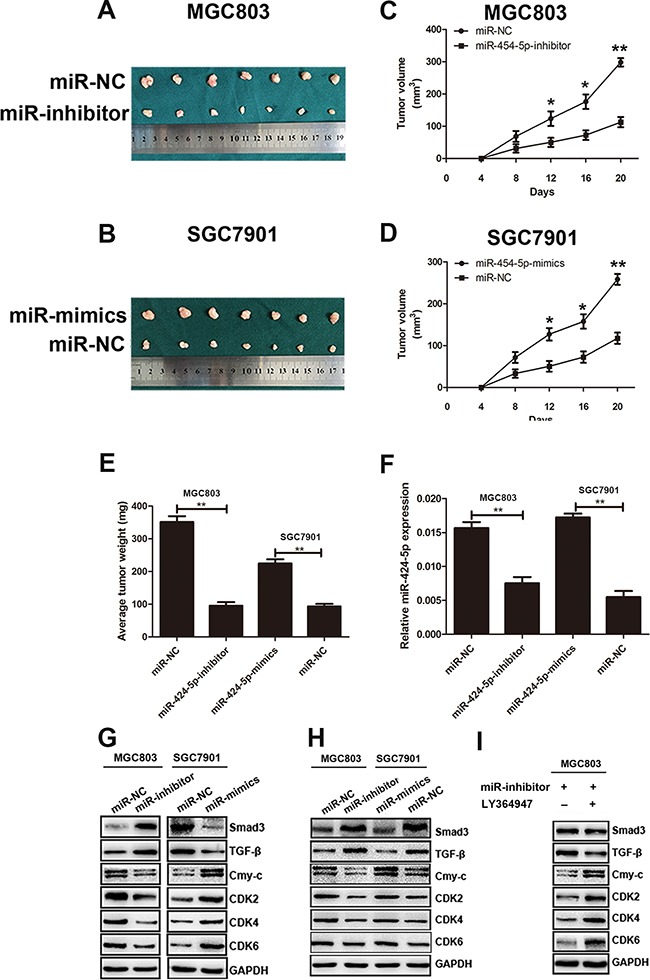
MiR-424-5p promoted tumor growth of GC cells in nude mice **A.** and **B.** Tumors were obtained from nude mice injected subcutaneously with cells transfected with miR-424-5p inhibitor and miR-424-5p mimics. **C.** and **D.** growth curve of tumor volumes. **E.** The average tumor weight of nude mice(*P<0.05). **F.** The expression levels of miR-424-5p in samples collected from nude mice. **G.** The protein expression levels of TGF-β, Smad3, c-Myc, CDK2, CDK4 and CDK6 in samples collected from nude mice were measured by western blotting. **H.** The protein expression levels of TGF-β, Smad3, c-Myc, CDK2, CDK4 and CDK6 in GC cells were measured by western blotting. **I.** The protein expression levels of TGF-β, Smad3, c-Myc, CDK2, CDK4 and CDK6 in MGC803 cells treated with LY364947 were measured by western blotting.

In order to investigate the effects of miR-424-5p expression on tumor growth in nude mice, MGC803 cells (2×106 cells in 200μL PBS) transfected with miR-424-5p inhibitor lentivirus and MGC803 cells transfected with negative control were injected subcutaneously into nude mice respectively. SGC7901 cells transfected with miR-424-5p mimics lentivirus and SGC7901 cells transfected with negative control were also injected subcutaneously into nude mice respectively. Compared with control, tumor volume and weight showed a significantly decrease in miR-424-5p-inhibitor-treated group. However, tumors in miR-424-5p-mimics-treated group showed the opposite affection (Figure 7a-7e). We also detected the expression of miR-424-5p in samples collected from nude mice by qRT-PCR. As shown in Figure 7f, the expression of miR-424-5p was decreased in miR-424-5p-inhibitor-treated group, while it was shown the opposite effect in miR-424-5p-mimics-treated group. In addition, the expression of Smad3 and other downstream targets of TGF-β were determined by western blotting. We found that the expression level of Smad3 and TGF-β was increased in miR-424-5p-inhibitor-treated group. However, the expression of c-Myc, CDK2, CDK4, CDK6 was decreased in the same group. It was shown the opposite results in miR-424-5p-mimics-treated group (Figure 7g).

### MiR-424-5p regulated TGF-β signaling pathway through Smad3

To further explore the mechanism how miR-424-5p induced loss of Smad3 promote the proliferation of GC cells both *in vitro* and *in vivo*, the TGF-β, Smad3, C-myc, CDK2, CDK4, CDK6 protein levels in GC cells were determined by western blotting. As shown in Figure 7h, the expression levels of Smad3 and TGF-β were significantly increased in MGC803 cells transfected with miR-424-5p inhibitor lentivirus compared with control. However, the expression levels of c-Myc, CDK2, CDK4, CDK6 were decreased in the same cells. In contrast, Smad3 and TGF-β proteins were down-regulated in SGC7901 cells transfected with miR-424-5p mimics lentivirus. Whereas the protein levels of c-Myc, CDK2, CDK4, CDK6 showed the increased expression in the same cells compared with control. To verify the role of TGF-β in the signaling pathway mediated by miR-424-5p, MGC803 cells transfected with miR-424-5p inhibitor lentivirus was treated by the TGF-β signaling pathway inhibitor LY364947 for 24h. There was no obvious difference on the expression of Smad3 between these two groups. In addition, the expression of TGF-β was decreased in cells which was added the LY364947. However, the downstream targets of TGF-β such as c-Myc, CDK2, CDK4, CDK6 showed the opposite effects in cells treated by LY364947 (Figure 7i). Taken together, these results suggested miR-424-5p might regulate TGF-β signaling pathway through Smad3, which resulted in the cell cycle proteins alteration.

## DISCUSSION

Gastric cancer is a common disease that causes cancer-related deaths worldwide [6]. Despite many efforts have been made to improve the diagnosis and treatment of GC, the prognosis of advanced GC patients is still poor [16, 17]. It was reported that miRNAs may act as either oncogenic factors or tumor suppressors depending on the tumor types and their targeted genes in human cancers [18, 19]. A lot of miRNAs have been reported to be associated with GC progression [6]. For instance, miR-137 inhibits cell growth and induces apoptosis through inactivating CDC42 in GC cells [20]. MiR-129 was down-regulated and leaded to over-expression of SOX4 in primary gastric cancers [21]. MiR-27b-3p suppressed cell proliferation through targeting ROR1 in gastric cancer [22].

Smad3 protein belongs to the SMAD gene family, which is a modulator of TGF-β signaling pathway. Smad3 plays a important role in cancer progression [23]. Transforming growth factor(TGF-β) signaling pathway was reported to be associated with a series of cellular processed [24]. Many studies have been focused in understanding how TGF-β signals modulate cell cycle [25]. An important role in the TGF-β signaling is the inhibition of c-Myc expression. TGF-β inhibits protein expression of c-Myc and cyclin D leading to inhibition of cyclin-dependent kinase (CDK) activities that drive the progression through G1 phase of the cell cycle [26]. It has been reported that c-Myc could control cell cycle entrance by regulating several factors participating in G1/S or in G2/M transitions [27].

In this study, our data revealed that miR-424-5p was up-regulated in gastric cancer. Over-expression of miR-424-5p could promote gastric cancer proliferation. Additionally, miR-424-5p could down-regulate Smad3 expression through binding to the 3'UTR of Smad3 mRNA. Up-regulation of Smad3 could reverse the promoting effect on GC cell proliferation of miR-424-5p. We also found that miR-424-5p promotes proliferation of gastric cancer cells by targeting Smad3 through TGF-β signaling pathways. In general, our findings provide a new prospective on the molecular therapy targets on gastric cancer treatment. However the exact mechanism how miR-424-5p promoted GC cell proliferation was not fully understood, it still needs us to pay more attention on it.

## MATERIALS AND METHODS

### The specimens and cell lines

We collected GC tissues and adjacent normal tissues from the patients who were performed radical gastrectomy in The First Affiliated Hospital of Nanjing Medical University. The written informed consent was obtained from all the patients or their relatives. This study was approved by Ethical committee of Nanjing Medical University.

All human GC cell lines MGC803, BGC823, SGC7901, AGS, HGC27 and human normal gastric epithelial cell line (GES-1) were purchased from Cell Center of Shanghai Institutes for Biological Sciences (Shanghai, China) and were cultured in RPMI-1640 medium supplemented with 10% fetal bovine serum at 37°C in a humidified atmosphere with 5% CO_2_, The TGF-β inhibitor LY364947 was purchased from Abcam (BRISTOL, UK).

### Quantitative real-time PCR

Total RNA was extracted from tissues and cells using Trizol (Takara, Japan) according to manufacturer's protocol and cDNA was synthesized using Primescript RT reagent (Takara, Japan). Relative expression level of miR-424-5p was normalized to the expression level of U6 and Smad3 expression was normalized to β-actin. The PCR reactions were performed using a 7500 Real-Time PCR System (Applied Biosystems, USA). The primers used in this study were as follows: has-miR-424-5p, forward:5'CAGCAGCAATTCATGTGTTTTGAA3';U6, forward:5'CTCGCTTCGGCAGCACA3';Smad3, forward:5'AGAAGACGGGGCAGCTGGAC3', reverse:5'GACATCGGATTCGGGGATAG3';β-actin, forward:5'GCATCGTCACCAACTGGGAC3' reverse: 5'ACCTGGCCGTCAGGCAGCTC3'.

### Western blotting

The protein was extracted from GC cells and tissues and transferred to polyvinylidene difluoride (PVDF) membranes. The transferred membranes were blocked in 5% non-fat powdered milk for 2h and incubated with primary antibodies overnight at 4°C. The membranes were then incubated for 2h in secondary antibodies at room temperature. The primary antibodies used in this study were as follows: Smad3 (diluted 1:500, Abcam), TGF-β, c-Myc, CDK2, CDK4, CDK6, GAPDH (diluted 1:1000, Cell Signaling Technology). GAPDH was used as an internal control.

### Immunohistochemistry

The immunohistochemistry method was performed as described preciously [28]. Smad3 (diluted 1:250, Abcam) was used as primary antibodies.

### Lentivirus production and transduction

The lentiviral vector containing Smad3 DNA sequence, lentiviral vector containing Smad3 siRNA hairpin sequence (LV-shSmad3), miR-424-5p mimics and inhibitor lentivirus was constructed by GenePhama (Shanghai, china). Target cells (2×10^5^) were infected with 1×10^6^ lentivirus transducing units in the presence of 1ug/ml polybrene.

### Luciferase reporter assay

The luciferase reporter assay method was performed as described preciously [29]. The 3'-untranslated regions (UTR) of Smad3 containing the wild or mutated miR-424-5p binding sequences were synthesized by Genescript (Nanjing, China).

### Plate clone assay

500 cells were plated in 6-well plates for each plate and culture in RPMI-1640 medium containing with 10% FBS. When the colony was obviously after nine days, the plates were then washed with PBS and stained with Giemsa for 15 min. All experiments were performed in triplicate.

### Cell cycle assay

The transfected cells were digested with trypsin and centrifuged at 1200 rpm for 5 min and washed twice in PBS. Afterwards, 4 ml ice-cold 75% ethanol was added in this solution to fix cells overnight. The centrifuged cells were then added with 500ul PI staining solution and incubated for 30 min. The distribution of cells was analyzed by a FACS Calibur flow cytometer with Cell Quset software (BD Biosciences)

### CCK8 assay

2000 cells were plated in 96-well plates and cultured in RPMI 1640 medium containing 10% FBS for 6 days. Cell Counting Kit-8 (CCK8) was used to evaluate the cell proliferation. In general, 10ul CCK8 solution was added to each plate and cells were incubated for 2 h in 37°C. The cell viability was revealed by the absorbance which was measured at 450 nm.

### Tumor xenograft in animals

4 weeks old female BALB/c nude mice were purchased from the Animal Centre of Nanjing Medical University. 2×106 cells in 200μL PBS were subcutaneously injected into each mice. Care of experimental animals was conducted according to the guidelines of the Nanjing Medical University Institutional Animal Care and Use Committee. Tumor growth was evaluated with calipers every 4 days. The volume of the tumor was calculated using the formula: Tumor volume=length×width^2^×0.5

### Statistical analysis

The data were expressed as mean ±standard deviation. The statistical analyses were performed using Student's *t* test (two-tailed) with the Social Sciences (SPSS) software version 19.0. Categorical data were evaluated by the X^2^ test. The data were considered significant when* P<0.05, **P<0.01, or ***P<0.001.
