# Correction: miR-424-5p promotes proliferation of gastric cancer by targeting Smad3 through TGF-β signaling pathway

**DOI:** 10.18632/oncotarget.17876

**Published:** 2017-05-15

**Authors:** Song Wei, Qing Li, Zheng Li, Linjun Wang, Lei Zhang, Zekuan Xu

**Present:** The current funding acknowledgment information is incorrect.

**Correct:** The proper funding information appears below. The authors sincerely apologize for this oversight.

**FUNDING**

This work was supported by the National Natural Science Foundation Project of International Cooperation (NSFC-NIH, 81361120398); the National Natural Science Foundation of China (81272712, 81572362); the Priority Academic Program Development of Jiangsu Higher Education Institutions (PAPD, JX10231801); the Program for Development of Innovative Research Team in the First Affiliated Hospital of NJMU; 333 Project of Jiangsu Province (BRA2015474).

Original article: Oncotarget. 2016; 7:75185-75196. doi: 10.18632/oncotarget.12092

